# Methodology for Integrating Mineral-Impregnated Carbon Fibers as Reinforcement in Fine Filament 3D Concrete Printing

**DOI:** 10.3390/ma19040786

**Published:** 2026-02-18

**Authors:** Tobias Neef, Marko Butler, Viktor Mechtcherine

**Affiliations:** Institute of Construction Materials, Dresden University of Technology, 01069 Dresden, Germany

**Keywords:** 3D concrete printing (3DCP), digital fabrication, mineral impregnated carbon fibers (MCF), non-metallic reinforcement

## Abstract

Mineral-impregnated carbon fibers (MCF) represent an advanced non-metallic reinforcement material offering high structural efficiency in concrete elements. Carbon fibers are impregnated with a suspension of ultrafine cement and microsilica and processed into rovings, providing high strength, design flexibility, and excellent bonding to concrete. In their freshly impregnated state, MCF exhibit high flexibility and can be deposited in any geometries, making them particularly suitable for complex structures manufactured using innovative processes such as 3D concrete printing (3DCP). Despite many advancements in reinforcement strategies for 3DCP, there is a lack of a simultaneous continuous corrosion-resistant reinforcement strategy. This is to be achieved by directly integrating freshly manufactured, still flexible MCF as longitudinal reinforcement of extruded concrete strands. Various modifications in MCF processing and print head modification are being investigated. This study highlights the potential of freshly impregnated MCF to improve structural continuity and automation due to their high flexibility. After modification, initial mechanical tests are carried out on printed MCF-reinforced concrete strands in comparison to cast speciments. These results are discussed and supplemented by visual findings from computer tomography. Although the mechanical performance of the printed specimens remains inferior to that of the cast specimens, as confirmed by CT analyses, the results demonstrate the feasibility of an effective method for simultaneous and continuous reinforcement of concrete during the 3D printing process.

## 1. Introduction

The construction industry faces significant challenges due to rising demands for construction work alongside a shortage of skilled labor [1,2]. Digitization presents a viable solution, enabling automation and robotics to enhance construction processes, boost productivity, reduce costs, and improve working conditions [3]. These advancements make the industry more attractive to skilled workers. Among the most promising innovations is 3D concrete printing (3DCP) [4].

3DCP utilizes specialized machines and concrete mixtures to fabricate structural elements and entire buildings based on digital planning data. Compared to traditional methods, this technology offers greater precision and efficiency, allowing for faster production and the realization of customized, intricate designs [5,6,7].

However, integrating 3D printing into construction also presents challenges, particularly in incorporating reinforcement into the digitally controlled additive manufacturing process for concrete structures [8].

Various approaches exist for reinforcing 3D-printed concrete [9,10,11,12]. For the simultaneous deposition of both concrete strands and continuous reinforcement, three techniques have been proposed: (1) using steel cables or chains [13,14,15,16], (2) integrating non-metallic fibers [17,18,19,20], and (3) utilizing meshes or two-dimensional textiles [21,22,23,24]. Each of these methods requires modifications to the print head, while allowing for fully automated reinforcement integration.

The article focuses on the use of non-metallic fibers, particularly mineral-impregnated carbon fibers (MCF), a novel type of concrete reinforcement developed at TU Dresden. In addition to carbon-based systems, silicon carbide (SiC) fibers have been proposed in the literature as an alternative non-metallic reinforcement, as they can be directly used without additional impregnation, potentially simplifying processing routes [25]. Utilizing corrosion-resistant carbon fibers allows for a very thin concrete cover. MCF exhibits excellent adhesion to concrete due to the material compatibility between the impregnation suspension and the concrete matrix [26]. This strong bond is maintained even at high temperatures, unlike polymer-bonded carbon fibers [27]. While SiC-based fibers offer high chemical and thermal stability, their higher cost and limited availability currently restrict their application in large-scale construction processes. Since MCF is highly formable in its freshly impregnated state, it can be processed and shaped more easily compared to metallic cables or chains, allowing for tight bending radii and high degrees of reinforcement. Additionally, MCF has a lower environmental impact and is more cost-effective than polymer-impregnated carbon fibers [28]. The integration of MCF can be interpreted as a material-level strategy to enhance post-cracking dissipation and damage control in concrete, in line with enriched concrete concepts proposed in the literature [29].

Continuous, freshly impregnated MCF reinforcement has been successfully integrated into relatively large strands of stiff concrete using a large print head operated by a gantry robot [18,30]. However, incorporating MCF into narrow strands of softer concrete using a slender print head manipulated by a 6-axis industrial robot proved to be highly challenging due to the following reasons:

- The fine filament printing process requires soft-consistency concrete, as it must be pumped through a narrow rubber hose to the nozzle. This softer fresh concrete lacks the strength to effectively pull and carry the fiber into the extruded strand through volumetric flow.
- Increasing the reinforcement ratio reduces the available volume of concrete, further limiting its ability to pull and embed the MCF.
- Despite imposing a uniform nozzle speed via the robotic arm, slight deviations in movement were observed due to physical and technical constraints set by the robot’s controller. These minor speed fluctuations resulted in either overfeeding, causing blockages in fiber deposition, or underfeeding, leading to excessive tension and fiber extraction from already deposited concrete strands. Although a software-based synchronization of fiber delivery speed with the robot arm movement was implemented, a hardware-based buffer at the fiber deposition station was ultimately required to resolve these issues.

In general, it can be concluded that integrating MCF into concrete strands requires pushing the MCF into the concrete, a stark contrast to other processing techniques for freshly impregnated (or untreated) carbon fibers, where fibers are typically pulled, as shown in references [31,32]. Since freshly impregnated MCF exhibits very low structural stiffness under compressive loads—comparable to a wool thread—it cannot simply be pushed into a concrete strand. Although the flexibility of MCF is advantageous for 3DCP, it presents challenges in ensuring its consistent integration into the process.

This article explores various approaches to address this challenge, specifically investigating physical or structural modifications of the fiber aimed at increasing its stiffness to facilitate integration into the concrete flow. The suggested solutions include variations in the fiber deposition method and corresponding modifications of the nozzle and the fibers. Ultimately, the most successful approach is presented and discussed, supported by heuristic analyses of the print results.

## 2. Materials and Setup

### 2.1. Fine Grained Concrete for 3D Printing

**Figure 1:**
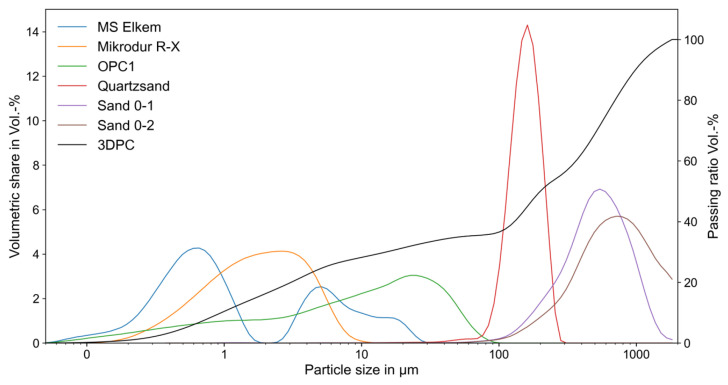
Particle size distributions of the individual components, along with the summarized passing ratios for 3D printed concrete (3DPC).

The composition of concrete heavily relies on the availability of raw materials, especially in 3D concrete printing where the choice of materials is crucial for adjusting particle size distribution. This distribution, combined with mixing water, significantly affects the concrete’s rheological behavior in its fresh state. To enhance pumpability and extrudability, Elkem’s Microsilica Powder 971, Thamshavn in Norwaywas selected as the finest ingredient. To bridge the particle size gap between Microsilica Powder and ordinary Portland cement CEM I 42.5 R from Holcim, Dyckerhoff’s Lägerdorf in Germany ultrafine ground cement Microdur R-X is used, with 95% of particles smaller than 6 µm. The particle distributions of the individual components are shown in Figure 1. These three components ensure a consistent increase in the particle size distribution in the binder paste. The sand component includes BCS 413 quartz fine sand, ranging from 0.06 to 0.2 mm, and locally sourced sands with grain sizes from 0 to 1 mm and 0 to 2 mm. These sand fractions contribute to achieving a densely packed system with a relatively low water demand and the desired rheological behavior of the fresh concrete mixture, including its buildability. The detailed composition of the printable fine-grained concrete used in this investigation is outlined in Table 1.

Once all the materials are dry-mixed, water is gradually added until a water-to-binder ratio of 0.42 is achieved. To attain a consistency suitable for additive manufacturing, superplasticizer MasterRheobuild 30 is added until the flow spread reaches 17.5 cm on the Haegermann table after 15 compaction strokes, as per [33]. The resulting concrete exhibits an average compressive strength of 88.0 MPa (with a standard deviation of 7.5 MPa) and an average flexural strength of 6.1 MPa (with a standard deviation of 0.6 MPa), as determined by a 3-point bending test on molded mortar prisms measuring 1600 × 40 × 40 mm^3^ according to DIN EN 12390-5 [34].

### 2.2. Fiber Impregnation Process for MCF

The fiber material used for producing mineral-impregnated carbon fibers (MCF) was Tenax-J/E STS40 E23 carbon fibers from Teijin Carbon Europe GmbH, Wuppertal in Germany. According to the manufacturer, each single carbon filament exhibits a tensile strength of 4300 MPa and a modulus of elasticity of 250 GPa. The carbon roving consists of 48,000 individual filaments and has a fineness of 3200 tex. The mineral impregnation suspension used in this study was adapted from previous research on MCF [30], drawing from various studies [9,19,27]. This suspension includes two ultrafine cements mixed with water and superplasticizer, and synergistically combined with a micro silica suspension, as detailed in Table 2. The suspension’s water-to-binder ratio was maintained at 0.8 to ensure a flowable yet robust mixture. Due to the intense grinding of the cements, 95% of the particles are smaller than 6 µm, allowing the mineral particles to effectively penetrate between the individual carbon filaments, each approximately 7 µm in diameter.

The homogeneous suspension of all particles was achieved by a thorough 5 min mixing process at 2500 rpm using an intensive single-shaft mixer, followed by an additional 2 min mixing process at 6500 rpm in the same device. The suspension’s viscosity was adjusted with a naphthalene-sulfonate-based superplasticizer MSH FLÜSSIG, yielding a Marsh Funnel [35] run-out time of approximately 30 s.

To impregnate the fibers, they were guided in a bath equipped over five rollers to ensure penetration of the particles between the carbon filaments by deflecting the roving over the rollers. Afterwards, the excess impregnation material was removed by a nozzle and returned to the impregnation bath. To bundle the individual carbon filaments, the MCF was wrapped with cotton yarn after impregnation. The MCF was then wound onto a spool, which was later attached to the print head. A detailed description of this impregnation process is provided in [19,30]. When fabricating MCF, a theoretical fiber volume content of about 35% is achievable, although practical trials achieved around 30%. The impregnated carbon roving (fresh MCF) was carefully wound onto an aluminum spool with a diameter of 16 cm. This spool was then attached to the print head assembly, allowing for seamless integration of the fresh MCF into the 3D printing nozzle.

To assess MCF’s mechanical performance in its hardened state, fresh MCF was stretched onto a rack to form 60 cm-long rebars. These cylindrical rebar geometries were tested as described in [30]. In displacement-controlled uniaxial tension tests at a speed of 1 mm/min, the tensile strength was measured at 2.15 MPa, and the failure strain was 10.6 per thousand.

### 2.3. Manipulator System

**Figure 2:**
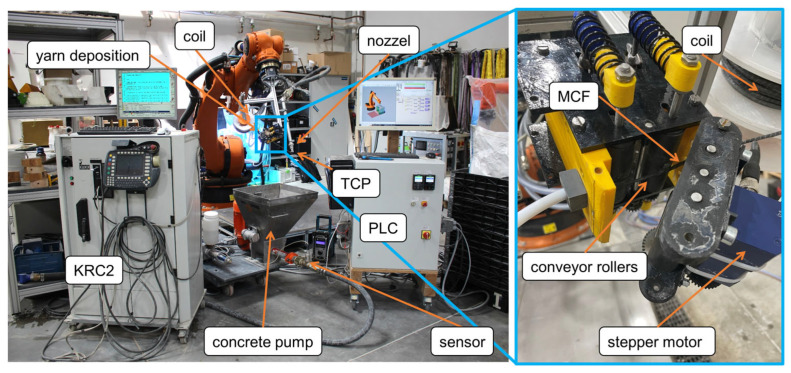
The **left** side shows the printing setup, while the **right** side displays the detailed fiber deposition in the blue box.

The 3D printing system comprises several key components, illustrated in Figure 2:

A progressive cavity pump by Knauf PFT GmbH and Co.KG, Iphofen in Germany for handling grain sizes up to 3 mm ensures continuous concrete delivery. A low-energy vibrator at the inlet hopper facilitates the uninterrupted flow of fresh concrete to the pump without manual assistance.

- A digital pressure sensor installed between the pump and the hose halts the pump if concrete pressure exceeds 20 bar, detecting potential blockages and enhancing safety.
- A 15 m-long hose with a nominal diameter of DN25 transports concrete from the pump to the nozzle.
- A nozzle mounted on a robot’s arm shapes the pumped concrete. Adjacent to the nozzle, the fibers feeding unit and spool are affixed to the robot’s arm end plate. This unit, comprising several rubber rollers driven by a stepper motor, feeds fibers from the spool to the nozzle.
- A six-axis industrial robot, KR 240-2 2000 by KUKA Deutschland GmbH, Augsburg in Germany, with a nominal payload of 240 kg and a maximum reach of 2.7 m, moves the assembly of the nozzle, hose, spool, and feeding unit precisely. The robot follows commands from the KRC2 controller, which processes the pre-generated printing path.
- A second control cabinet houses electronics for controlling the mortar pump, stepper motors, and sensors, and for integrating signals from the KRC2 controller.

The robot’s controller (KRC2) dictates the movement speed at the tool center point (TCP) via a 10 V signal processed by a programmable logic controller (PLC) within the TwinCAT 3 automation software by Beckhoff Automation GmbH & Co. KG, Verl in Germany. This PLC setup not only controls the mortar pump but also aligns the pump’s delivery speed with the TCP’s movement, effectively coordinating the nozzle’s operation.

## 3. Integration of Fresh MCF in the 3D Printing Process

### 3.1. Modification of the Nozzle—Location of the MCF Insertion

**Figure 3:**
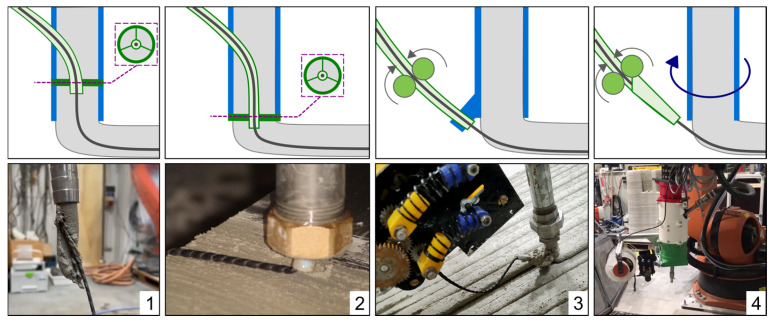
Modification of the nozzle: (**1**) supply of MCF into the concrete volume before the nozzle tip, (**2**) centric insertion of MCF shortly after the nozzle tip, (**3**) deposition of MCF before the nozzle with overprinting, (**4**) deposition of MCF before the endlessly rotating nozzle with overprinting.

The integration of freshly impregnated MCF into 3D printing entails modifying the nozzle to longitudinally insert the MCF at specific locations in the concrete strand with a cross-section of 20 mm × 10 mm (as used in this investigation). Figure 3 illustrates the various approaches that have been tested:

(1) MCF Insertion Before Nozzle Tip

MCF is inserted into the concrete volume before reaching the nozzle tip, drawn by the friction between the flowing concrete and MCF from the spool. The freshly impregnated MCF passes through a PTFE hose, positioned centrally, to reduce curvature and friction before merging with the concrete. This method suits stiffer concrete mixes and offers the flexibility to pause and resume reinforcement. However, the positioning within the concrete cross-section remains imprecise, often pushing the MCF to the edge due to internal pressures.

(2) Centric Insertion After Nozzle Tip

The PTFE hose extends slightly beyond the nozzle tip, releasing MCF directly into the freshly extruded concrete, ensuring central placement within the strand. This method maintains the fibers’ centrality during nozzle rotation but creates cavities around the MCF due to fixation disturbances and rapid concrete setting, leading to potential MCF debonding.

(3) MCF Deposition Before Nozzle

MCF is laid down just before the nozzle by a short, slightly bent PTFE hose, ensuring that it is sandwiched centrally between two concrete strands. This independent reinforcement placement allows for precise MCF positioning but weakens the bond between consecutive concrete layers and between the MCF and the base strand, especially with rapid-setting concretes or extended layer times.

(4) MCF Deposition with Rotating Nozzle

This approach decouples the rotation of the nozzle and concrete hose, allowing the nozzle to align the MCF with the printing path while the hose rotates independently, avoiding entanglement. This enhances geometrical freedom and simplifies path programming. While initial prototypes show increased maintenance and complexity, further refinement is expected to mitigate these drawbacks.

Each method’s effectiveness varies based on the concrete viscosity, reinforcement positioning, and operational mechanics, necessitating ongoing refinement to optimize MCF integration in 3D concrete printing.

### 3.2. Modification of the MCF and the Fibers Feeding

The tests conducted in Section 3.1 demonstrated that the fresh concrete used in this study is too soft to securely anchor the fresh MCF. Consequently, insufficient tensile forces can develop in the MCF to reliably overcome the friction between the MCF and the PTFE tube. When the print head moves along small radii, the MCF is pulled out of its intended position within the concrete strand and, in the worst case, entirely out of the concrete. To address this issue, a fibers feeding unit was introduced. This unit pulls the fresh MCF from the spool, safely absorbing the forces generated in the process. While it was also intended to push the fresh MCF through the PTFE hose toward the concrete, this concept often fails because the stiffness of fresh MCF under compressive loads is very low. Typically, carbon fibers are processed under tension [31].

**Figure 4:**
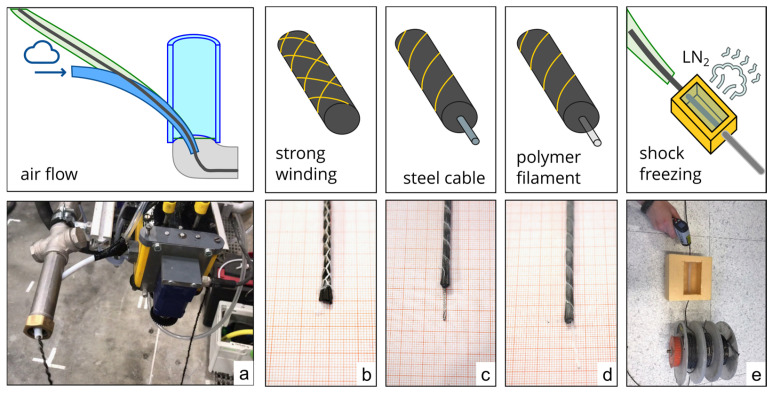
Modifications of the MCF and fibers feeding: (**a**) continuous airflow, (**b**) strong winding, (**c**) insertion of a steel cable, (**d**) insertion of a polymer filament, and (**e**) shock freezing.

Section 3.2 presents various modifications to the MCF and its processing, aimed at ensuring the robust and reliable delivery of fresh MCF during pushing. These measures must not compromise the key properties that make MCF suitable for 3D printing, such as flexibility and bond strength. Figure 4 illustrates the investigated measures.

(a) Conveying the filament with a continuous airflow

Approach: An airflow is introduced into the PTFE hose via nozzle modification 1, oriented in the direction of fibers feeding (see Figure 4a). This airflow is intended to facilitate fibers conveyance and mitigate clogging. In the event of an obstruction, the increased air pressure pushes the blockage toward the hose outlet. Additionally, the airflow is designed to reduce friction between the MCF and the PTFE hose.

Discussion: The feeding of the MCF through the nozzle without concrete was significantly improved, with minimal risk of fibers clogging. The system demonstrated stability in response to minor disturbances and compensated for uneven robotic movement. Moreover, filament integration at the tool center point (TCP) of the nozzle was possible. However, challenges remain, such as the high energy consumption required to maintain air pressure throughout printing and the need to exhaust the airflow without disrupting the formation of homogeneous concrete strands. Furthermore, conveying MCF using compressed air caused loosening of the freshly impregnated filament bundle, compromising the bond intended to develop via mineral impregnation after curing. Under load, the interaction between individual filaments was disrupted, reducing the overall performance of the MCF.

(b) Increasing stiffness through strong winding

Approach: By tightly winding the freshly impregnated MCF with a cotton thread, the friction between individual filaments is increased. This reduction in slip between filaments enhances the stiffness of the filament bundle under compression loads.

Discussion: The stability of MCF conveyance improved due to the increased stiffness. Tests on non-impregnated rovings yielded successful results and provided additional protection against filament loosening. However, since no suitable technical means were available to achieve dense winding after impregnation, tests were conducted on uncoated fibers. Only impregnated fibers can provide high performance, rendering this approach impractical. An alternative attempt involved first winding the fibers and then impregnating them; however, effective impregnation requires filament spreading for uniform particle migration [12], which was hindered by the tight winding.

(c) Increasing stiffness by inserting a steel cable into the MCF

Approach: After impregnating the carbon fibers with a mineral suspension, a steel cable (~1 mm in diameter) was inserted into the filament bundle. The impregnated carbon filaments and steel cable were then shaped into a round cross-section, secured by winding with a thin cotton thread.

Discussion: The increased stiffness provided by the steel cable improved the conveyance of the entire MCF and facilitated smooth integration into the concrete line. Additionally, the steel cable contributed to load transfer and could be detected in the concrete using inductive methods. When a stainless-steel cable was used, corrosion risks were mitigated. However, limitations arose due to the large bending radius of the cable. Tight curves or turns could not be printed without the cable–MCF bundle being pushed out of the concrete strand.

(d) Increasing stiffness by inserting a polymer filament

Approach: Instead of a steel cable, a polymer filament was used as a stiff core within the MCF bundle. The processing followed the same method as in Approach (c).

Discussion: Initially, very thin polymer filaments (0.4 mm) were tested, but they lacked sufficient stiffness for consistent MCF conveyance. Thicker polymer filaments (1–1.2 mm) resulted in excessive bending radii, causing similar issues to those in Approach (c). The best results were obtained with a polymer filament of 0.7 mm in diameter, which provided sufficient stiffness for smooth conveyance while maintaining a small bending radius. Additionally, polymer filaments are corrosion-resistant, lightweight, flexible, and cost-effective compared to stainless-steel cables. The flexibility can be adjusted by selecting an appropriate diameter, enabling printing of smaller radii compared to stainless steel. However, unlike a steel cable, the polymer filament does not provide additional functional benefits such as load-bearing capacity.

(e) Temporary increase in stiffness through shock freezing

Approach: To improve the transport properties of the freshly impregnated MCF, it was frozen. The water in the mineral impregnation suspension solidified into ice, significantly increasing fibers’ stiffness while halting the hydration reaction of reactive mineral particles. Two implementation methods were tested:

Freezing the entire spool with pre-impregnated MCF.

By guiding the fibers through a basin filled with liquid nitrogen (LN_2_) to freeze it immediately before entering the nozzle, due to its low mass and thermal capacity, the MCF thawed instantly upon contact with the extruded concrete (~25 °C), restoring its original flexibility.

Discussion: Freezing the entire spool at −18 °C temporarily increased stiffness. A solid aluminum spool was manufactured as a thermal buffer, preserving the frozen state for over two hours in lab conditions. However, individual filaments froze together and adhered to the spool, causing breakage when unwound.

For the second approach, a liquid nitrogen bath with a PTFE transmission tube was constructed (see Figure 4e). The unfrozen MCF was guided through this cold tunnel without direct contact with LN_2_. At a printing speed of 10–20 cm/s, temperatures around −20 °C were measured on the MCF surface upon exit. This approach increased stiffness without additional materials such as steel or polymer filaments. However, challenges included filament damage in the first implementation, high technical complexity in the second, and significant costs associated with LN_2_ supply. Due to process control challenges, financial implications, and stringent safety protocols, further pursuit of this method was deemed unjustified.

### 3.3. Successful Modification of Nozzle, MCF, and Fibers Feeding

Ultimately, the combination of nozzle modification (3) and fibers modification (c) proved successful. In this setup, the filament is deposited through a short PTFE hose positioned ahead of the nozzle, allowing the concrete strand to overprint it. The short PTFE hose ensures precise positioning of the reinforcement. The MCF is integrated into the concrete without tension. Additionally, the free section between the hose and the fibers deposition allows for the formation of a loop, which compensates for movement discontinuities.

**Figure 5:**
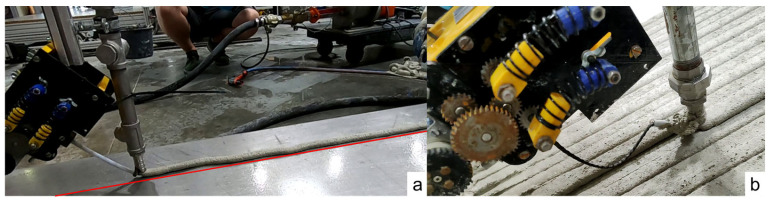
(**a**) Printing process with 0.4 mm support filament, (**b**) final setup for print tests.

An extension, referred to as modification 4, is proposed to render the nozzle endlessly rotatable. Reinforcing the MCF with a support filament ensures seamless conveyance; however, determining the appropriate diameter is crucial. Printing tests with diameters of 0.4 mm, 0.7 mm, and 1.0 mm revealed that 0.4 mm was too soft to effectively push the filament, while 1.0 mm was too stiff, causing the filament to be displaced laterally from the concrete strand. Due to the stiff fibers, the printed concrete strand was not straight but exhibited a snail-like pattern, as shown in Figure 5a.

The best results were achieved using a polyamide support filament with a diameter of 0.7 mm from Decofil Mamutec. The printing process with this final setup is shown in Figure 4b.

In a print test using this setup, an initial layer of unreinforced concrete was deposited. This layer had a thickness of 9 mm and dimensions of 800 × 600 mm^2^. To form this layer, 30 adjacent strands were laid, each with a width of 20 mm. Subsequently, a second layer was deposited, in which MCF was integrated into each strand. This layer also had a thickness of 9 mm, resulting in a total printed structure thickness of 18 mm.

Although this method is well-suited for manufacturing complex reinforced structures, the focus of this digital production process was on horizontal geometries to facilitate sample removal for mechanical material testing. These samples will undergo heuristic testing, with the results presented in the following section.

**Figure 6:**
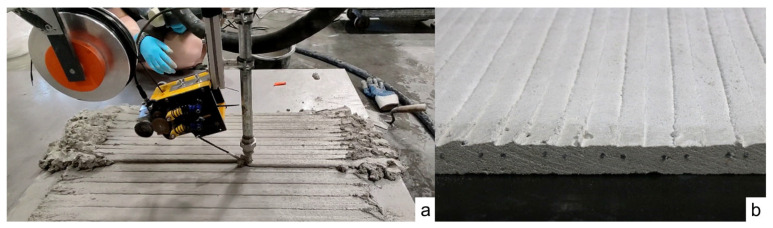
(**a**) Digital fabrication of a reinforced slab for mechanical investigation. (**b**) Cross-Section of the slab after hardening.

The production of the second layer of the slab is shown in Figure 6a, while Figure 6b illustrates the position of the MCF reinforcement within the slab.

## 4. Mechanical Properties

### 4.1. Uniaxial Tension Tests on MCF and Its Modifications

The tensile strength of the mineral-impregnated fibers was tested to evaluate the changes in mechanical performance based on the applied modifications (cf. Section 3.2). Although only the MCF modification with a 0.7 mm support filament was used successfully for printing, additional tests were conducted on MCF with 0.4 mm and 1.0 mm support filaments, as well as on MCF briefly frozen with liquid nitrogen after impregnation. Conventionally produced MCF was tested as a reference. A total of 10 samples per type were analyzed.

**Figure 7:**
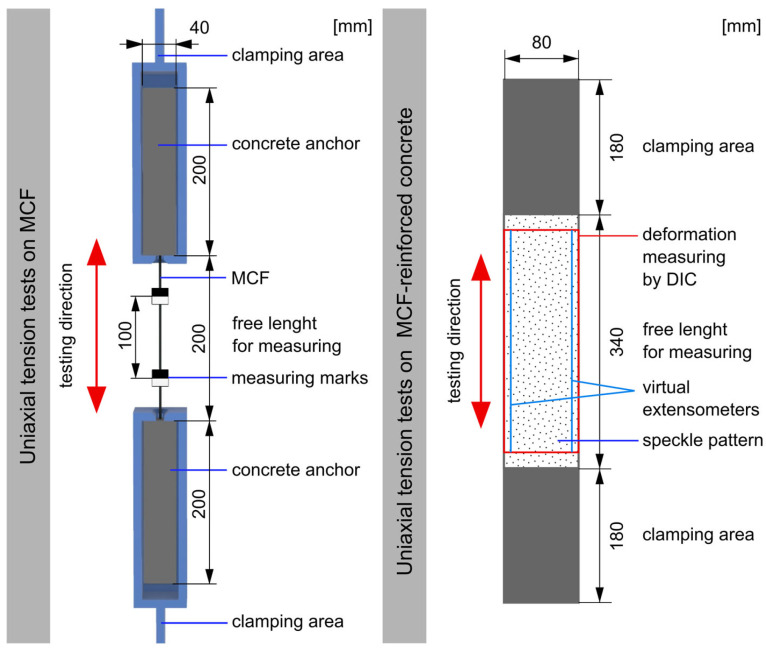
Experimental setup for tensile tests on MCF and on printed and cast MCF-reinforced specimens.

Due to the sensitivity of the fibers and impregnation to lateral pressure applied by direct clamping in the tensile testing machine, the MCF samples were embedded in concrete blocks at both ends; see Figure 7. These blocks ensured gentle load transfer during testing. The test setup and procedure are described in detail in [30,36]; here, only the key aspects are summarized.

Tensile testing was performed using a hydraulic universal testing machine (Instron 8802) equipped with a 20 kN load cell, at a testing speed of 1 mm/min. Deformation measurements were conducted with an electro-optical extensometer (200XR), which recorded displacement using black–white markers glued 100 mm apart on the MCF.

From the experimental data, tensile strength, failure strain, and Young’s modulus were calculated (see Table 3). Regardless of filament diameter, modifications involving a support filament showed no significant deterioration or improvement compared to the reference. All measured values fell within a similar range, within standard deviation. Additionally, brief shock freezing had no notable effect on the mechanical performance of the MCF.

### 4.2. Uniaxial Tension Tests on Deposited and Cast MCF-Reinforced Concrete Specimens

#### 4.2.1. Sample Preparation and Test Setup

The tensile behavior of cast and 3D-printed concrete reinforced with MCF was investigated through preliminary uniaxial tensile tests. These tests, conducted on a limited series of specimens, aimed to investigate the mechanical behavior of MCF-reinforced concrete produced using the proposed integration process with modification 3c. For this purpose, five specimens with dimensions of 18 × 80 × 700 mm^3^ were extracted from the two-layer printed slab described in Section 3.3. The longitudinal axes of the specimens were aligned parallel to the MCF orientation. To enable a comparative assessment between the 3D printing approach and conventional manufacturing methods, eight additional specimens with identical dimensions were produced by casting. Each subgroup of specimens was reinforced with four MCF embedded in the fresh state.

The tensile tests were conducted using a hydraulic universal testing machine (Instron 8501). The tensile load was transferred from the machine to the sample by full-face clamping at both ends, using hydraulic jaws. The clamping area was set at 180 mm on both ends, leaving a free test length of 340 mm in the center of the sample. Within this free section, a 300 mm gauge length was designated for deformation measurement; see Figure 7.

All specimens were stored under standard climate conditions (20 °C, 65% RH) after fabrication and tested at a concrete age of 28 days. The tensile tests were conducted under displacement control, with a constant crosshead displacement rate of 1 mm/min. Throughout testing, both the applied displacement and resulting force were continuously monitored and recorded.

Deformation along the measurement range was recorded using two virtual extensometers. Additionally, surface deformations and crack development were monitored at 1 Hz using a digital image correlation (DIC) system (ARAMIS, Zeiss-GOM). This dual-camera system enabled three-dimensional displacement recording, ensuring accurate calculations of deformations, crack widths, and strains within the measurement field. To ensure precise measurement, the measurement field was ground and prepared with a stochastic black–white speckle pattern before testing.

#### 4.2.2. Results of the Tensile Tests

**Figure 8:**
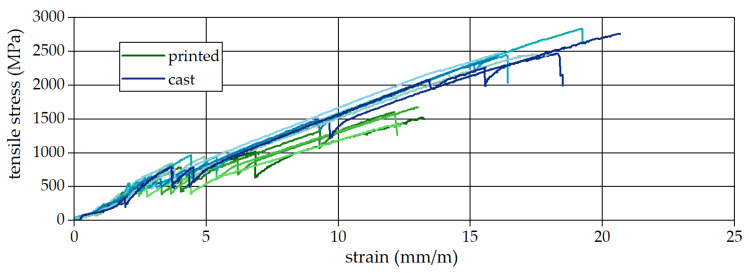
Stress–strain curves obtained from tensile tests.

The results are presented in a stress–strain diagram for all samples (see Figure 8). Deformation data were obtained from DIC measurements up to 90% of the ultimate strain and, in parallel, from the crosshead displacement converted into axial strain. Additionally, cracks were automatically counted using the DIC system. In the diagram, the curves of the cast samples represented are shown in blue, while the curves of the printed samples are shown in green. Both variants exhibit a linear stress–strain relationship, which is characteristic of carbon-reinforced concrete composites.

Table 4 presents the results from uniaxial tension tests conducted on both cast and printed specimens. The average tensile strength of the cast samples reached 106.8% of the tensile strength of non-embedded MCF (see Table 3). The cast samples exhibited a higher tensile strength, averaging 2470 MPa. This exceptionally high strength is hypothesized to result from the superior bond between the MCF and the concrete matrix.

In contrast, the printed samples had a significantly lower tensile strength, averaging 1560 MPa. This reduction is attributed to the geometric misalignment of the MCF during the digital fabrication process, which is considerably more challenging than in conventional production methods. The observed reduction in the tensile strength and strain capacity of printed specimens can also be attributed to impaired bond conditions and load transfer mechanisms, phenomena that are known to govern nonlinear pull-out behavior in reinforced cementitious composites [37]. Furthermore, the printed specimens exhibited earlier failure, with an average failure strain of 12.7 mm/m, which is close to the failure strain measured in the tensile tests of non-embedded MCF (10.1 mm/m), compared to the cast specimens with 17.4 mm/m.

Another difference is further reflected in crack formation, as the cast samples exhibited twice as many cracks per meter compared to the printed specimens: thirteen cracks per meter versus six cracks per meter, respectively.

### 4.3. Visual Inspection

Visual examinations of the composite were conducted to elucidate the findings described in Section 4.2. Traditional sample preparation methods for visual inspection, such as sawing and splitting, often result in the loss of critical information regarding the delicate bond zone between MCF and concrete. Consequently, non-destructive examination techniques were employed. Computed tomography (CT) proved highly effective, enabling visualization of both the bond interface and the orientation of the fiber within the concrete [30,38,39].

For the CT analysis, small cores were extracted along the MCF axis from the center of the printed slab, each with a diameter of 15 mm and a length of 70 mm. Similarly, cores of identical dimensions were obtained from the undamaged clamping regions of the cast specimens used in the uniaxial tension tests. The drill cores were positioned to ensure that the MCF aligned with the core’s central axis.

CT scans were performed using a Mikro-CT XPRESS system (ProCon X-Ray GmbH, Sarstedt, Germany). During scanning, samples underwent a 360° rotation, captured in 2960 increments with an exposure time of 0.25 s per increment. Reconstruction was executed using X-AID software version 2022.5.0 (MITOS GmbH, Munich, Germany), with automatic determination of center shift and beam hardening for each sample. Post-reconstruction, the scanned volumes were processed for image analysis.

**Figure 9:**
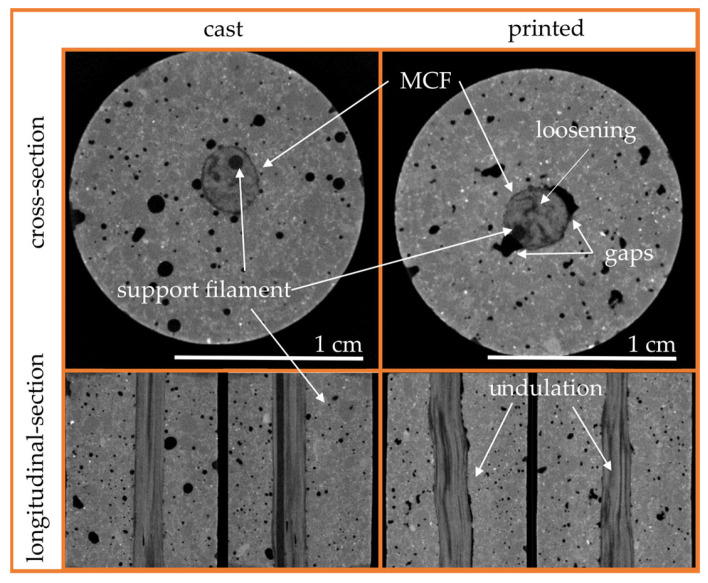
Computed tomography scans of cast and printed specimens.

Figure 9 presents cross-sectional and longitudinal views from the CT reconstructions of both the cast and printed specimens, offering detailed insights into the interface between the MCF and the surrounding concrete.

In the cast sample (Figure 9 Left), no significant voids were observed between the concrete and MCF, though small pores occasionally adhered directly to the fiber’s surface. The cross-section of the roving clearly reveals the support filament, tightly enclosed by the surrounding MCF. Longitudinal sections demonstrate an undisturbed, linear alignment of individual MCF filaments, ensuring optimal structural integrity and efficient force transmission, as all filaments are evenly stretched under load. Additionally, the contact surface between the fibers and the concrete is free of gaps, indicating a strong bond. Notably, the longitudinal section shows the support filament fully embedded within the MCF.

Conversely, the printed specimen’s cross-section (Figure 9 Right) reveals a closed contact zone between the MCF and concrete at the top and bottom, but distinct gaps are evident on the sides. These gaps likely result from the manufacturing process; during overprinting, complete encapsulation and embedment of the MCF by the deposited concrete was not fully achieved. A lower-viscosity printing matrix might reduce these gaps and enhance bonding. The shading effects observed in the reinforcement suggest the loosening of individual filaments, with the support filament located at the edge of the roving. Longitudinal sections confirm these observations, displaying loosened and undulating filaments.

The misalignment of carbon fibers adversely affects the composite’s mechanical properties, as previously discussed in Section 4.2. The primary causes of these irregularities are believed to be the small radius of the spool on which the fiber was wound, the tension-free placement of the MCF prior to overprinting, and the pushing mechanism used during fiber deposition, which leads to the loosening of impregnated filaments.

Identifying these deficiencies facilitates the development of optimization strategies to enhance the structural integrity and mechanical properties of 3D-printed MCF-reinforced concrete. Potential improvements include modifying the printing matrix viscosity to improve the MCF–concrete interaction, optimizing fiber deposition techniques to prevent filament loosening, and enhancing spool design to minimize pre-printing misalignment.

## 5. Conclusions

Various approaches for integrating freshly impregnated mineral carbon fibers (MCF) into 3D concrete printing have been investigated, with a particular focus on overcoming the low structural stiffness of fresh MCF and their susceptibility to misalignment. These challenges have been effectively addressed by incorporating a supportive polymer filament into the MCF during the impregnation process. This polymer filament ensures stable transport of the continuous reinforcement during printing. The polymer-based support filament is light, cost-effective, corrosion-resistant, and allows for smaller bending radii. As a result, it enhances design flexibility without compromising the mechanical performance of the MCF.

The modification of the nozzle ensures the precise, tension-free placement of the fiber in front of the nozzle opening, which facilitates concrete placement and reinforcement integration. A free length between the fiber deposition unit and the nozzle provides a buffer zone for the conveyed MCF, reducing process disturbances caused by robot motion discontinuities.

With the identified modifications, printing tests were carried out and a planar MCF-reinforced plate was printed. Specimens were cut from the plate and initial uniaxial tensile tests were performed. The printed specimens were compared with conventionally manufactured cast samples.

The results show that the modification presented achieved the effective integration of MCF into 3DCP. However, when comparing tensile strength, the digitally fabricated specimens only achieve an average of around 60% of the load-bearing capacity of cast specimens. The reason for this is the insufficient alignment of the individual carbon filaments within the MCF, which was demonstrated by CT examinations of cast and printed specimens. Despite the need for optimization to improve the mechanical properties, an effective method for the simultaneous and continuous reinforcing of concrete during the 3D printing process was developed.

## 6. Outlook

Future work will focus on extending continuous MCF-reinforced concrete printing from simple flat geometries to more 3D structures. This will require improved path planning strategies and coordinated extrusion and MCF integration. In addition, the potential adaptation of the support filament will be investigated to enable selected functional enhancements, such as accelerated curing or basic sensing capabilities. Overall, these developments aim to further improve the applicability and robustness of continuous reinforcement strategies in 3DCP.

## Figures and Tables

**Table 1:** Composition of a fine grained concrete for 3D printing [kg/m^3^].

Composition	Grain Size	Volume [kg/m^3^]
Micro silica	d95 < 14 µm	14
Micro cement Mikrodur R-X	d95 < 6 µm	395
OPC I 42.5R	d95 < 9 µm	335
Fine quartz sand	0.06/0.2 mm	230
Sand	0–1 mm	370
Sand	0–2 mm	795
Water		279
Superplasticizer		17
w/b		0.42

**Table 2:** Composition of the mineral suspension for impregnation of carbon fibers (masses in g for 1 L suspension) [30].

Micro Silica d90 < 0.7 μm	Micro Cement 1 d95 < 6 μm	Micro Cement 2 d95 < 11 μm	Superplasticizer	Water	w/b
345.4	345.4	345.4	31.1	493.3	0.8

**Table 3:** Results of the uniaxial tensile test on MCF.

	Tensile Strength		Strain at Failure		Young’s Modulus	
	MPa		m/mm		GPa	
MCF	AV	SD	AV	SD	AV	SD
Reference	2149	166	10.6	1.0	194	13
Support filament 0.4 mm	2206	153	9.4	1.7	226	35
Support filament 0.7 mm	2316	306	10.1	2.2	195	16
Support filament 1.0 mm	2257	99	10.1	2.2	199	21
Shock freezing	2002	21	11.0	3.5	217	40

**Table 4:** Results of the uniaxial tensile test on concrete reinforced with MCF.

	Tensile Strength		Failure Strain		Number of Cracks per Meter	
	MPa		mm/m		-	
Method	AV	SD	AV	SD	AV	SD
Cast	2470	207	17.4	2.1	13	5
Printed	1560	87	12.7	0.5	6	2

## Data Availability

The original contributions presented in this study are included in the article. Further inquiries can be directed to the corresponding author.
